# A Case of Acute General Peritonitis, Treated by Morphine in Large Doses

**Published:** 1891-10

**Authors:** George A. Himmelsbach

**Affiliations:** House-Physician Buffalo General Hospital


### Clinical Report.

### A CASE OF ACUTE GENERAL PERITONITIS, TREATED BY MORPHINE IN LARGE DOSES.—RECOVERY.

By GEORGE A. HIMMELSBACH, M. D.,
House-Physician Buffalo General Hospital.

Bessie L., aged ten years, entered hospital July 15th with a history as follows: About ten days before entering indulged freely in watermelon, and at same time ate more than half of a quart of peanuts. From that time until July 21st, she had recurring attacks of pain in abdomen. On this date she was first seen by a physician, who reports as follows: Temperature, 101½°; pulse, 145; great tenderness over whole of abdomen, the slightest movement causing her to shriek from pain.

The doctor diagnosticated peritonitis, and at once put her on the saline treatment, magnesii sulphas was given in two and a half drachm doses every three hours. This, however, was only kept up for three days, when the stomach rejected the salts. Opium was then resorted to, and was given in small doses until she entered the hospital.

At time of entering, patient's abdomen was very much distended, with marked tympanites, and she was suffering excruciating pain. Temperature, 100°; pulse, 134; respiration, 24. Under Dr. Stockton's directions the child was immediately put upon the opium treatment, as recommended by Alonzo Clark. Morphine in doses of one-quarter grain each was given hypodermically as frequently as needed to keep the child in a semi-narcotized condition, and as nearly free from pain as possible. My instructions were not to allow the patient to come out from the effects of the drug, hence close observance was instituted, and just as soon as she showed evidence of this, such as flexion of thighs, restlessness, with pulse becoming slow, soft and full, the drug was repeated.

It soon became evident that the dose given was not sufficient. I accordingly gradually, but cautiously, increased the dosage until July 30th the highest point was reached, the maximum amount given in twenty-four hours being twenty-four grains, as follows: 9.00 A. M., grs. iv.; 3.00 P. M., grs. iv.; 4.30 P. M., grs. iv.; 7.40 P. M., grs. iv.; 10.30 P. M., grs. iv., and 3.40 A. M., grs. iv., all hypodermically.

It will be observed that the child had by this time attained an extraordinary tolerance for the drug, the amount which was given at the onset of the malady and with the desired effect would, if given now, be followed by no reaction at all.

This may appear to some as very heroic and, perhaps, dangerous treatment, but it is in this very fact that the whole secret of this treatment lies. The drug must be given for its effect, regardless of the amount. It may be well to note here that at no time did the respirations fall below fifteen per minute, and ranged from this point to twenty-eight per minute.

The total amount of morphine given from July 16th to August 3d was 170 grains, all hypodermically, and the largest dose given at one time was on July 31st, grs. v. From this time on, the child showing signs of improvement, the dose was gradually reduced until gr. $\frac{1}{4}$ was reached, this being continued for a few days, and finally stopped. Ice bags were then applied, which relieved the pain greatly.

In addition to the opium, other agencies were used to relieve the distension. Hot fomentations were applied every fifteen minutes to half an hour, but with little relief. Aqua camphoræ in ʒss doses was given every half hour for some time, but without much effect.

July 20th, the tympanites became very great, and on account of the increased tension, there appeared spots of discoloration, the respirations were somewhat labored, the heart was so displaced that the apex beat could be felt in the second intercostal space; it was plain that something more radical must be done. The rectal tube was used. A soft, flexible tube was introduced with no little difficulty to the extent of three feet; it was then allowed to remain in situ for twenty-four hours, after which it was removed for twelve hours to give the parts rest. This was done alternately for periods of twelve hours, and with the happiest result.

July 26th, the tympany having disappeared, the tube was removed.

Up to July 30th, the bowels had moved but twice, and both stools were very small. On this date in palpating the abdomen, an indurated mass was felt in the left iliac region, fecal accumulation was suspected, and a mild enema, consisting of ol. terebin, ol. gossipii, and soap-suds, was given, resulting in free evacuation and disapperance of the tumor. From this time on, the child improved rapidly. Tonics, such as albuminate of iron, champagne, beef juice, were given freely to build up the much-reduced system. The patient was in condition to leave the hospital about August 20th, but owing to the unsanitary and poor hygienic surroundings of her home, it was deemed safer to keep her under treatment and observation at the hospital for some time thereafter.

